# Knowledge of HIV/AIDS among older adults (50 years and above) in a peri-urban setting: a descriptive cross-sectional study

**DOI:** 10.1186/s12877-019-1335-4

**Published:** 2019-11-11

**Authors:** Reindolf Anokye, Enoch Acheampong, Amy Budu-Ainooson, Edmund Isaac Obeng, Emmanuel Tetteh, Yvonne Sabby Acheampong, Curtis Edward Nettey-Marbell

**Affiliations:** 1 Centre for Disability and Rehabilitation Studies, Department of Community Health, Kwame, Nkrumah University of Science and Technology, Kumasi, Ghana; 2 School of Medical and Health Sciences, Edith, Cowan University, Joondalup, WA Australia; 3 School of Public Health, Department of Health Education and Promotion, Kwame Nkrumah University of Science and Technology, Kumasi, Ghana; 4 Methodist University College, Accra, Ghana; 5 Garden City University College, Kumasi, Ghana; 6 Department of Clinical and Health Information Sciences, University of Cincinnati, Cincinnati , Ohio, USA

**Keywords:** Knowledge, Signs and symptoms, Prevention, Transmission, HIV/AIDS, Ghana

## Abstract

**Background:**

In the absence of vaccine or cure, public knowledge about Human Immune Virus (HIV) is a central tool for curbing HIV epidemic. This study sought to assess the knowledge of HIV among older adults (50 years and above) at the Methodist Faith Healing hospital, Ankaase, Ghana.

**Methods:**

Using a descriptive study design, older adults (50 years and above) who visited the Ankaase Methodist Faith Healing hospital were randomly sampled for the study. A structured questionnaire was administered to collect data which was analyzed quantitatively using Statistical Package for Social Sciences (SPSS version 16.0).

**Results:**

A total of 100 respondents who were aged 50 to 68 (54 ± 2.3) were recruited. Most of the respondents had average knowledge of the mode of HIV transmission (62%) as well as HIV prevention (58%) and signs and symptoms of HIV (60%). HIV status was significantly associated with HIV knowledge among older adults as HIV positive respondents were 2.25 times more knowledgeable in terms of signs and symptoms, mode of transmission and prevention of HIV [AOR (95% CI) 2.25(1.02–8.68)].

**Conclusion:**

Most older adults (50 years and above) have average knowledge of the mode of transmission, prevention as well as signs and symptoms of HIV. The National Commission for Civic Education should collaborate with various key stakeholders to educate older adults on issues related to HIV/AIDS.

## Background

Public knowledge of Human Immune Virus (HIV) could be a key tool in the management of HIV [1] and in many countries where HIV prevalence is high, an increase in comprehensive correct knowledge of HIV could help reduce its incidence [2].

In 2013, the Joint United Nations Programme on HIV/AIDS (UNAIDS) reported that about 4.2 million older adults were living with HIV globally [3]. The prevalence has increased over the years in all WHO regions with significant increases recorded in Central and Western Europe as well as the United States of America and Canada [3, 4]. According to the Centers for Disease Control and Prevention (CDC), approximately 45% of patients diagnosed with HIV in 2014 were adults aged 50 years and above [5]. In 2016, it was reported that out of 39,782 new cases of HIV, 6812 (17%) were diagnosed among adults aged 50 years or above in the United States [5]. New HIV cases among older adults across the WHO European region [6] and in specific European Economic Areas (EEA) have also been reported [7, 8]. Furthermore, 31% of study participants who were adults aged 50 years or older in a Swiss HIV Cohort were reported to be HIV positive [9]. In 2003, 10.4% of new HIV cases were recorded among adults aged 50 years or above in Western Europe increasing to12.9% in 2007 [6]. Similarly, the percentage of older adults who were diagnosed with HIV infections in Eastern Europe increased over the same time frame [10]. In sub-Saharan Africa, several HIV cases among older adults have been reported [10] and a Gap report [11] highlighted that among more than two million people aged 50 and above living in Sub-Saharan Africa, about 60% of them are living with HIV and AIDS.

According to Negin and Cumming [10], the five (5) countries with the highest number of older adults living with HIV in sub-Saharan Africa were Mozambique, Nigeria, South Africa, Zambia and Zimbabwe. In 1999, 2.6% of men and women aged 55 to 70 years were identified as living with HIV and AIDS in Cameroon [12]. In Dar es Salaam in Tanzania, HIV prevalence of 15% among those aged 55 or older have been recorded [13] and 175 cases among people aged 55 years and older were recorded from 1990 to 1996 in Congo [14].

An increased incidence of HIV among older adults have been generally reported [15] with a steadily rising prevalence over the years. However, many older adults do not consider themselves to be at risk of HIV and perceive it as an illness for younger people [16]. Stall and Catania [17], reported that older adults are one-sixth as likely to use condoms during sex and one-fifth as likely to have HIV test when compared to at-risk people in their twenties. Orel, Spence and Steele [18], also reported that older adults, particularly older women are at greater physiological risk for HIV transmission because they may engage in new relationships in which unprotected sex is more likely given the absence of pregnancy concerns.

At the Methodist Faith Healing hospital in Ankaase, 319 new HIV (incident) cases were recorded between 2013 and 2015. Out of this number, 175 (55%) were associated with older adults (50 years and above). This implies that the prevalence of HIV among older adults within the study area is very high. Even though the incidence of HIV among older adults has been increasing over the years, older adults are largely overlooked when it comes to HIV treatment, management and prevention programs [11]. Therefore, documenting the knowledge of HIV could be a key force in shaping policy formulation and interventions aimed at influencing health-seeking behaviour and prevention of HIV among older adults. This study seeks to assess the knowledge of HIV among older adults (50 years and above) at Ankaase, Ghana.

## Methods

This study was conducted at the Methodist Faith Healing hospital which is a non-profit and non-governmental organization that seeks to provide holistic health care to all patients. The facility provides services such as maternity, laboratory, in-patient services, surgery, nutrition, accident and emergency, X-ray, public health, and mortuary services. The facility currently serves as a referral hospital for two main districts within Ashanti Region (Afigya Kwabre and Kwabre).

A descriptive study design using a quantitative approach was employed in assessing the knowledge of HIV among older adults (50 years and above) at Ankaase Methodist Faith Healing hospital. The study design appropriately helped the investigators to gain insights into the problem and provided a range of causes and alternative options for a solution to the research problem.

The study population comprised of older adults (50 years and above) who were HIV positive and those who were not. Older adults (50 years and above) were targeted because little is known about older adults living with HIV in sub-Saharan Africa. Also, 319 new HIV cases were recorded at the study site between 2013 and 2015 and 175 (55%) older adults were involved. There is also the likelihood that people aged 50 years or older will require specialized care if they contract HIV and it’s important therefore that their knowledge about HIV is assessed to aid in preventive strategies and interventions.

A closed-ended questionnaire (A File 1) specifically designed for this study was used to collect quantitative data. The questionnaire comprised of four subsections containing questions on the demographic characteristics of respondents; mode of transmission of HIV; prevention of HIV as well as signs and symptoms of HIV. The language for the questionnaire was originally English but was translated into several local languages during administration. The local languages were Akan, Hausa and Ewe which were the dominant languages spoken by respondents. The translation was done by experts who were very conversant with these languages and one (1) translator was recruited for each particular language. The investigators were mindful of the usage of words due to the sensitive nature of the subject under investigation and the target population.

Knowledge of HIV was determined based on the average scores of respondents (total of correctly answered questions for each sub-section divided by the number of questions which was later summed and divided by 3) in questions on the mode of transmission as well as prevention and signs and symptoms of HIV. An average score of < 50 was rated as poor knowledge; an average score of 50 to 75 was rated as average knowledge and an average score of 75 and above was rated as good knowledge. The average scores of < 50, 50 to 75 as well as 75 and above were parameters that were set by the investigators to conclude that the respondent’s knowledge was low, high or average. The Statistical Package for Social Sciences (SPSS version 16.0) was used to analyze the data.

A simple random sampling technique was used to select the respondents for the study. The sample size was determined using Yamane [19] simplified formula for calculating sample sizes. This formula was used to calculate the sample size using 95% confidence level (The value of (1-α) in Standard Normal Distribution ***z***-table, which is 1.96 for 95%) and a precision level/sampling error or margin of error of 0.05 or 5% which is the generally acceptable margin of error for social research [20]. The investigators, therefore, claim that there is a 95% chance that the confidence interval that was calculated contains the average of all older adults (50 years and above) who visited the Methodist Faith Healing hospital with a margin of error of 5%. The 95% confidence level implies that 19 out of 20 times we conduct this survey; the results would land within our margin of error. The 5% margin of error suggests that if we surveyed all 131 older adults, the results could differ with a score of minus 5% or plus 5% from its original score. The Yamane [19] formula was used to calculate for the sample using the Equation below;

$$ n=\frac{N}{1+N{(e)}^2} $$

*n* represents the sample size to be attained, *N* is the population size, and *e* is the level of precision. Records at the hospital revealed that 131 older adults had visited the hospital within the previous 6 months preceding the study. The following sample was attained using the Yamane [19] simplified formula;

$$ {\displaystyle \begin{array}{c}\mathrm{N}=131\\ {}1+N{(e)}^2=1+131{(.05)}^2\\ {}n=\frac{131}{\begin{array}{l}1+131{(.05)}^2\\ {}\mathrm{n}=96\end{array}}\end{array}} $$

A 10% non-respondent rate was expected and therefore 9 were added to 96 to achieve a minimum sample of 105 respondents. In selecting the respondents, a set of even and odd numbers were written on pieces of papers for them to pick on each day. Daily, respondents who picked even numbers were selected and the questionnaires were administered. Data were collected within a period of 2 months and 4 research assistants were employed. The research assistants and the investigators were able to reach out to the 105 respondents within two months to collect data. Older adults who were HIV positive visited the facility each month for the Anti-Retroviral therapy and/or to take their medications, so the investigators were able to reach out to each one of them within the two months without any difficulties. The others who were not HIV positive visited the facility for various reasons and the investigators contacted them to collect data. The number of respondents who were contacted within the two months is representative of the entire population as all HIV patients are required to visit a healthcare facility each month for either Anti-Retroviral therapy or to take their medications. The facility’s status as a referral hospital for two main districts within the Ashanti Region also made it easier to have contacts with older adults who were not HIV positive.

An introductory letter was sent to the hospital Authorities for approval to conduct the study at the hospital. The administrators of the hospital approved the use of the facility for the study before the data collection commenced. The risk, purpose and potential benefits were explained to respondents before their involvement in the study and the investigators emphasized that all information would be treated as strictly confidential.

A pre-test of the questionnaires was conducted at The Kumasi South hospital which has the same characteristics as the Methodist Faith Healing hospital. Cronbach’s alpha test was used to measure the internal consistency of the instruments. Through this test, the investigators can explain and interpret the reliability of the instruments. According to Zikmund [21] and George and Mallery [22], the Cronbach’s alpha reliability coefficients smaller than 0.60 suggest poor reliability, the coefficients from 0.60 to 0.70 indicates fair reliability, the coefficients from 0.70 to 0.80 indicates good reliability and the coefficients that is larger than 0.80 is considered excellent. According to Jacobson [23], the coefficient of alpha should be at minimum 0.70 or more for applied research and 0.90 or more in clinical trials. The pretest was conducted among five (5) older adults who were conveniently sampled. The scores obtained after administering the pretest questionnaire was 0.84 for the mode of transmission of HIV, 0.89 for prevention of HIV and 0.85 for signs and symptoms of HIV. This suggests relatively high internal consistency and therefore the instruments were deemed reliable. An expert further evaluated the instruments for basic grammatical errors and typographical errors and amendments were effected.

The research committee at Kwame Nkrumah University of Science and Technology approved the study. Respondents were informed about the aim of the study and its scope and sought their consent to participate. Verbal consent was taken from respondents because a good number of them could not read or write. The respondent’s consent was recorded on a digital recorder/tape recorder and in the researcher’s notes. This was more appropriate as some of the respondents were either illiterate or were unlikely to sign a form because of cultural reasons. The process was approved by the ethics committee after further explanation. The identity of the study respondents was kept confidential throughout the study and to maintain anonymity, participants’ identity was not disclosed at any point in this study.

## Results

### Demographic characteristics

Table 1 shows the respondent’s demographic data and as shown in the table, a little over half of the respondents (57%) were males while a third (31%) were single. More than half (67%) were married and close to half (49%) were Akans. Traders were dominant (46%) as well as those with SSCE/A level (43%) education.

**Table 1:** Demographic Characteristics of respondents

Variable	Category	Frequency (*n* = 100)	Percentage
Age in years	50–55	61	61%
Gender	Male	57	57%
	Female	43	43%
Marital Status	Single	31	31%
	Married	67	67%
	Divorced/separated	1	1%
	Widowed	1	1%
Ethnicity	Akan	49	49%
	Ewe	17	17%
	Ga/Adangme	5	5%
	Gonja	11	11
	Dagomba	17	17
	Others	1	1%
Religion	Christian	55	55%
	Muslim	39	39%
	Traditionalist	5	5%
	No religion	1	1%
Occupation	Trader	46	46%
	Farmer	7	7%
	Apprentice	6	6%
	Student	16	16%
	Artisan	4	4%
	Civil servant	3	3%
	Unemployed	5	5%
	Hairdresser	13	13%
Level of Education	BECE/O level	38	38%
	SSCE/A level	43	43%
	HND/Bachelors	7	7%
	No formal Education	12	12%

### Knowledge of HIV/AIDS

As shown in Table 2, 77% of the respondents stated that HIV/AIDS could be transmitted through the transfusion of unscreened blood from an infected person; 89% of the respondents were of the view that HIV/AIDS is transmitted from an infected mother to a child during the process of childbirth whilst 76% of the respondents affirmed that HIV/AIDS could be transmitted by the sharing of blades and injection needles. A sizeable number (81%) indicated that HIV/AIDS could be transmitted through insect bites or domestic animal bites and more than half of the respondents (75%) were of the view that HIV/AIDS could be transmitted through unprotected heterosexual sex with many partners.

**Table 2:** Knowledge of HIV

Variables		%
HIV mode of transmission	Transfusion of unscreened blood Infected mother to a child during birth Sharing of blade/injection needles Insect bites or domestic animal bites Having unprotected heterosexual sex with many partners Sharing same room or bed with HIV positive person Having sex with a partner with an open injury on penis/vagina The body sweats from an HIV-positive person Incorrect/inconsistent use of a condom Oral sex Breastfeeding by HIV-positive mother	77% 89% 76% 81% 75% 79% 86% 56% 67% 45% 34%
HIV prevention	By consistent use of condoms By abstinence from sex totally By being faithful to my partner By keeping to one partner at a time By avoiding sharing toilets with people living with HIV/AIDS By avoiding shaking hands with people living with HIV/AIDS	92% 85% 68% 59% 23% 34%
Signs and symptoms of HIV	Chronic or intermittent diarrhoea An intermittent (continuous) fever around or above 38 °C Weight loss Night Sweats Swollen lymph nodes Fatigue/lethargy	67% 72% 98% 56% 8% 78%

*n* = 100

The consistent use of condoms had the highest mention (92%) among options for HIV prevention as shown in Table 2.

Moreover, almost all the respondents (98%) mentioned weight loss as a sign and symptom of HIV/AIDS. However, only a few (8%) knew that a swollen lymph node is a sign and symptom of HIV as shown in Table 2.

### Knowledge performance

The average knowledge performance as shown in Table 3 for the Mode of HIV transmission was 69.5; prevention was 60.16 and signs and symptoms were 63.16.

**Table 3:** Average knowledge performance

Performance	Mean	Standard Dev
Mode of transmission	69.5	7.6
Prevention Signs and Symptoms	60.2 63.2	9.3 11.1

**Fig. 1:**
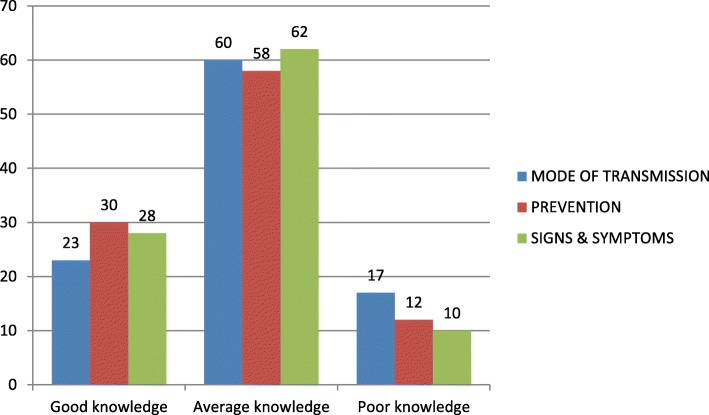
Knowledge of mode of transmission, prevention and signs and symptoms of HIV. Left: Good knowledge of the mode of transmission, prevention and signs and symptoms of HIV Middle: Average knowledge of the mode of transmission, prevention and signs and symptoms of HIV. Right: Poor knowledge of the mode of transmission, prevention and signs and symptoms of HIV. Results are expressed in percentage form.

The Fig. 1 shows that a little over half of the respondents (62%) had average knowledge on the mode of transmission of HIV while almost one-third (28%) had good knowledge and a tenth (10%) had poor knowledge of HIV mode of transmission.

Also, a little over half of the respondents (58%) had average knowledge of HIV prevention while about one-third (30%) had good knowledge of HIV prevention and about one-eighth (12%) had poor knowledge of HIV prevention.

As shown in Fig. 1, a section of the respondents had average knowledge (60%), good knowledge (23%) and poor knowledge (17%) of the signs and symptoms of HIV.

### Socio-demographic factors influencing HIV knowledge

Table 4 displays the Socio-demographic factors influencing HIV knowledge. As shown in the table, only HIV status (*p* = 0.012) influenced HIV knowledge among the respondents. Older adults who were HIV positive were 2.25 times more knowledgeable in terms of signs and symptoms, mode of transmission and prevention of HIV [AOR (95% CI) 2.25(1.02–8.68)].

**Table 4:** Socio-demographic factors influencing HIV knowledge

Variable	Knowledge of HIV score		Univariate	Multivariate*
	< 50	> 50	OR (95% CI)	AOR (95% CI)
Age				
50–55	31	30	1.00	1.00
56–60	10	8	0.49 (0.41–3.02)	1.21 (0.35–4.31)
61–65	5	10	2.61(0.17–2.78)	1.38 (0.04–2.29)
> 65	4	2	3.12 (1.98–21.0)	1.99 (0.12–2.01)
Gender				
Male	30	27	1.00	1.00
Female	23	20	4.23(3.11–4.87)	3.12(2.01–3.92)
Marital Status				
Married	37	30	1.00	1.00
Not married	13	20	10.8 (3.54–14.49)	3.03 (1.63–58.26)
Level of Education				
None	10	28	1.00	1.00
BECE/O level	22	21	1.08 (0.48–12.39)	1.62 (0.41–4.60)
SSCE/A level	4	3	1.18 (0.32–4.01)	2.00 (0.17–21.05)
HND/Bachelors	6	6	1.33 (0.81–1.92)	1.09 (0.14–1.78)
Employment Status				
Unemployed	3	2	1.00	1.00
Employed	45	50	8.21(0.12–2.18)	2.28(1.03–9.61)
Religion				
Christian	20	35	1.00	1.00
Muslim	19	20	3.11(1.13–4.18)	0.22 (0.18–2.95)
Traditionalist	2	3	1.15 (0.31–4.05)	2.01 (0.13–21.08)
No religion	0	1	1.68 (0.53–1.97)	1.15 (0.11–1.73)
HIV Status				
Not positive	12	38	1.00	1.00
Positive	27	23	2.51(3.10–20.19)	2.25(1.02–8.68)*

*Mutually adjusted, OR = Odds Ratio, CI = Confidence Interval, AOR = Adjusted Odds Ratio, 1.00 = Reference group, *p < 0.05

## Discussions

Behaviour change and increased comprehensive correct knowledge have been recently associated with the reduction of high HIV incidence and prevalence in many countries [2]. This study, therefore, explored older adult’s knowledge of HIV/AIDS and the socio-demographic factors influencing their knowledge. The findings revealed that 10% of respondents had poor knowledge of HIV mode of transmission, 12% had poor knowledge of HIV prevention and 17% had poor knowledge of the signs and symptoms of HIV. This lack of knowledge by a proportion of the respondents suggests that older adults need education in all possible ways of HIV prevention as well as modes of transmission and signs and symptoms. Similarly, a study among older African Americans reported a lack of knowledge about how HIV was transmitted [24].

The highest proportion of the respondents were categorized as having average knowledge of the mode of HIV transmission. Similarly, a study in Nigeria found that older adults were most knowledgeable about HIV/AIDS mode of transmission [25]. Also, most of the participants of this study knew that HIV could be transmitted from an infected mother to a child during birth corresponding with findings reported by Oschi, Nakalema and Oschi [25].

Peltzer [26] reported similar misconceptions about HIV/AIDS found in this study, and some of his respondents indicated that HIV could be prevented by avoiding sharing toilets with people living with HIV/AIDS (23%) as well as avoiding shaking hands with people living with HIV/AIDS (34%). This shows poor knowledge of HIV/AIDS generally among older adults and suggests that further education to improve their general knowledge of HIV and AIDS is needed. This will change their perception, and improve their understanding of the risk factors for HIV infection.

Furthermore, most of the respondents (89%) of this study knew that HIV could be transmitted from an infected mother to a child during birth, could be prevented by consistent use of condoms (92%) and that weight loss (98%) is a sign and symptom of HIV/AIDS. This is in line with a study that reported that most adults were knowledgeable about HIV [25] and GDHS report of 2003 stating that general knowledge about HIV transmission during pregnancy, delivery, and breastfeeding was relatively high and ranges between 69% and 75% among women [27].

Majority of the respondents of this study knew that the consistent use of a condom is a means of preventing HIV infection. This implies that consistent education on the use of condom as a means of preventing HIV has yielded fruits in Ghana. Total abstinence from sex as a means of preventing HIV was endorsed by 85% of the respondents. An earlier study reported higher endorsement for abstinence (95%), being faithful to one partner (90%), and lower endorsement for consistent condom use (82%) [28] as the best ways to prevent HIV. Also, a greater proportion of the respondents knew that having sex with an open injury on penis/vagina can lead to HIV/AIDS infection. Similarly, Dunkle et al. [28], reported that 90% of their respondents in a survey in Zambia knew that being faithful to one partner can prevent HIV. Though a lower proportion (68%) in this study knew that being faithful to one’s partner is a means of preventing HIV, it is still encouraging that respondents were aware that sticking to one sexual partner could prevent HIV infection. The difference in the proportion could be attributed to the high prevalence of HIV in Southern Africa which may have influenced the education on sticking to one sexual partner.

This study reports no significant relationship between gender of respondents and knowledge of HIV. However, Henderson [29] reported that 65% of women aged 50 and older had poor HIV knowledge and a high misperception of HIV transmission risk, with 63% believing HIV could be contracted through kissing. The findings of this study, therefore, contradict with that of Henderson’s.

Other studies have reported a relationship between low knowledge and increase in age. For instance, Hicks et al. [30] found that older patients score much lower on HIV knowledge tests. Abel and Werner [31] also found that people older than 45 had lower HIV knowledge scores. Wright et al. [32] reported that a third of their respondents who were older adults lacked knowledge on HIV and Maes and Louis [33] found a correlation between low knowledge and increase in age. This study did not report any significant relationship between age and knowledge of HIV/AIDS corresponding with findings of Ama, Shaibu and Burnette [34].

## Conclusions

Most of the respondents generally had average knowledge of the mode of transmission, prevention and signs and symptoms of HIV. However, the respondent’s knowledge of the mode of HIV transmission was higher than their knowledge of its prevention and signs and symptoms. Older adults who were HIV positive had more knowledge of the signs and symptoms, mode of transmission and prevention of HIV.

### Recommendations

The average knowledge of HIV/AIDS suggests that the Government of Ghana should increase efforts to educate older adults on issues related to HIV/AIDS. Programmes that seek to address the various misconceptions about HIV/AIDS transmission and clarify ways through which HIV can be transmitted as well as prevented and signs and symptoms that manifest when one has HIV/AIDS should be encouraged. Also, The National Commission for Civic Education should collaborate with various key stakeholders to provide HIV related education for older adults.

### Limitations

The study was limited to older adults in the Ashanti Region where the study was conducted and one facility where data was collected. The findings were however not compromised in any way by these limitations.

## Supplementary information

**Additional file 1.** Section a: socio demographic characteristics of respondents. Section b: knowledge of hiv/aids transmission. Section c: knowledge of hiv/aids prevention. Section d: knowledge of the signs and symptoms of hiv.

## Data Availability

The datasets used and/or analysed during the current study are available from the corresponding author on reasonable request.

## Abbreviations

AIDS: Acquired Immune Deficiency Syndrome
CDC: Centers for Disease Control and Prevention
EEA: European Economic Areas
GDHS: Ghana Demographic Health Survey
HIV: Human Immune Virus
SPSS: Statistical Package for Social Sciences
WHO: World Health Organisation
