# Supplementary material for: Knowledge of HIV/AIDS among older adults (50 years and above) in a peri-urban setting: a descriptive cross-sectional study
Source: BMC Geriatr. 2019 Nov 11;19:304. doi: 10.1186/s12877-019-1335-4 (PMC6849319; doi:10.1186/s12877-019-1335-4)
Supplement: Supplementary file 1 — Additional file 1. Section a: socio demographic characteristics of respondents. Section b: knowledge of hiv/aids transmission. Section c: knowledge of hiv/aids prevention. Section d: knowledge of the signs and symptoms of hiv. [file 12877_2019_1335_MOESM1_ESM.docx]

**QUESTIONNAIRE**

**SECTION A: SOCIO** - **DEMOGRAPHIC CHARACTERISTICS OF RESPONDENTS**

**INSTRUCTIONS: Please tick [√] where applicable**

1. Gender

a) Male [ ]

b) Female [ ]

3. What is your Marital Status?

a) Married [ ]

b) Separate [ ]

c) Divorced [ ]

d) Single [ ]

e) Widowed [ ]

4. What is your level of education?

a) No formal education [ ]

b) Primary education [ ]

c) Middle / JHS Education [ ]

d) SHS / Secondary Education [ ]

e) Tertiary education [ ]

f) Others (Specify)…………………….

5. Ethnicity

a) Akan [ ]

b) Ewe [ ]

c) Ga/Adangme [ ]

d) Other (specify) ……………………….

6. Religion:

a) Christian [ ]

b) Moslem [ ]

c) Traditionalist [ ]

**SECTION B: KNOWLEDGE OF HIV/AIDS TRANSMISSION**

| **HIV can be transmitted through:** | **True** | **False** |
| --- | --- | --- |
| 7. Unprotected sex with an infected partner(s) |  |  |
| 8. Transfusion of unscreened blood |  |  |
| 9.Infected mother to a child during birth |  |  |
| 10. Sharing of blade/injection needles |  |  |
| 11.Insect bites or domestic animal bites |  |  |
| 12. Having unprotected heterosexual sex with many partners |  |  |
| 13.Sharing the same room or bed with an HIV positive person |  |  |
| 14. Having sex with a partner with an open injury on penis/vagina |  |  |
| 15. The body sweats from an HIV-positive person |  |  |
| 16. Incorrect/inconsistent use of a condom |  |  |
| 17. Oral sex |  |  |
| 18. Breastfeeding by HIV-positive mother |  |  |

**SECTION C: KNOWLEDGE OF HIV/AIDS PREVENTION**

| **How can HIV infection be prevented?** | **True** | **False** |
| --- | --- | --- |
| 19. By consistent use of condoms |  |  |
| 20. By abstinence from sex totally |  |  |
| 21. By being faithful to my partner |  |  |
| 22. By keeping to one partner at a time |  |  |
| 23. By trusting God for protection, no matter how many persons one has sex with |  |  |
| 24. By avoiding sharing toilets with people living with HIV/AIDS |  |  |
| 25. By avoiding shaking hands with people living with HIV/AIDS |  |  |

**SECTION D: KNOWLEDGE OF THE SIGNS AND SYMPTOMS OF HIV**

| **What are some of the signs and symptoms of HIV** | **True** | **False** |
| --- | --- | --- |
| 26. Chronic or intermittent diarrhoea |  |  |
| 27. Immune system becomes very weak |  |  |
| 28. An intermittent (continuous) fever around or above 38°C |  |  |
| 29. Weight loss |  |  |
| 30. Night sweats |  |  |
| 31.Swollen lymph nodes |  |  |
| 32. Fatigue/lethargy |  |  |
| 33.Opportunistic infections |  |  |
